# The diagnostic value of nasal microbiota and clinical parameters in a multi-parametric prediction model to differentiate bacterial versus viral infections in lower respiratory tract infections

**DOI:** 10.1371/journal.pone.0267140

**Published:** 2022-04-18

**Authors:** Yunlei Li, Chantal B. van Houten, Stefan A. Boers, Ruud Jansen, Asi Cohen, Dan Engelhard, Robert Kraaij, Saskia D. Hiltemann, Jie Ju, David Fernández, Cristian Mankoc, Eva González, Wouter J. de Waal, Karin M. de Winter-de Groot, Tom F. W. Wolfs, Pieter Meijers, Bart Luijk, Jan Jelrik Oosterheert, Sanjay U. C. Sankatsing, Aik W. J. Bossink, Michal Stein, Adi Klein, Jalal Ashkar, Ellen Bamberger, Isaac Srugo, Majed Odeh, Yaniv Dotan, Olga Boico, Liat Etshtein, Meital Paz, Roy Navon, Tom Friedman, Einav Simon, Tanya M. Gottlieb, Ester Pri-Or, Gali Kronenfeld, Kfir Oved, Eran Eden, Andrew P. Stubbs, Louis J. Bont, John P. Hays

**Affiliations:** 1 Department of Pathology & Clinical Bioinformatics, Erasmus MC Cancer Institute, University Medical Center Rotterdam, Rotterdam, The Netherlands; 2 Division of Paediatric Immunology and Infectious Diseases, University Medical Centre Utrecht, Utrecht University, Utrecht, The Netherlands; 3 Department of Medical Microbiology and Infectious Diseases, Erasmus MC Cancer Institute, University Medical Center Rotterdam, Rotterdam, The Netherlands; 4 Streeklab Haarlem, Haarlem, The Netherlands; 5 MeMed, Tirat Carmel, Israel; 6 Division of Paediatric Infectious Disease Unit, Hadassah-Hebrew University Medical Centre, Jerusalem, Israel; 7 Department of Internal Medicine, Erasmus MC Cancer Institute, University Medical Center Rotterdam, Rotterdam, The Netherlands; 8 Noray Bioinformatics, Derio, Spain; 9 Department of Paediatrics, Diakonessenhuis, Utrecht, The Netherlands; 10 Department of Paediatric Respiratory Medicine, University Medical Centre Utrecht, Utrecht University, Utrecht, The Netherlands; 11 Department of Paediatrics, Gelderse Vallei Hospital, Ede, The Netherlands; 12 Department of Respiratory Medicine, University Medical Centre Utrecht, Utrecht University, Utrecht, The Netherlands; 13 Department of Internal Medicine and Infectious Diseases, University Medical Centre Utrecht, Utrecht University, Utrecht, The Netherlands; 14 Department of Internal Medicine, Diakonessenhuis Utrecht, Utrecht, The Netherlands; 15 Department of Respiratory Medicine, Diakonessenhuis Utrecht, Utrecht, The Netherlands; 16 Department of Paediatrics, Hillel Yaffe Medical Centre, Hadera, Israel; 17 Department of Paediatrics, Bnai Zion Medical Centre, Haifa, Israel; 18 Department of Internal Medicine A, Bnai Zion Medical Centre, Haifa, Israel; 19 Pulmonary Division, Rambam Health Care Campus, Haifa, Israel; Shandong Public Health Clinical Center: Shandong Provincial Chest Hospital, CHINA

## Abstract

**Background:**

The ability to accurately distinguish bacterial from viral infection would help clinicians better target antimicrobial therapy during suspected lower respiratory tract infections (LRTI). Although technological developments make it feasible to rapidly generate patient-specific microbiota profiles, evidence is required to show the clinical value of using microbiota data for infection diagnosis. In this study, we investigated whether adding nasal cavity microbiota profiles to readily available clinical information could improve machine learning classifiers to distinguish bacterial from viral infection in patients with LRTI.

**Results:**

Various multi-parametric Random Forests classifiers were evaluated on the clinical and microbiota data of 293 LRTI patients for their prediction accuracies to differentiate bacterial from viral infection. The most predictive variable was C-reactive protein (CRP). We observed a marginal prediction improvement when 7 most prevalent nasal microbiota genera were added to the CRP model. In contrast, adding three clinical variables, absolute neutrophil count, consolidation on X-ray, and age group to the CRP model significantly improved the prediction. The best model correctly predicted 85% of the ‘bacterial’ patients and 82% of the ‘viral’ patients using 13 clinical and 3 nasal cavity microbiota genera (*Staphylococcus*, *Moraxella*, and *Streptococcus*).

**Conclusions:**

We developed high-accuracy multi-parametric machine learning classifiers to differentiate bacterial from viral infections in LRTI patients of various ages. We demonstrated the predictive value of four easy-to-collect clinical variables which facilitate personalized and accurate clinical decision-making. We observed that nasal cavity microbiota correlate with the clinical variables and thus may not add significant value to diagnostic algorithms that aim to differentiate bacterial from viral infections.

## Introduction

Lower respiratory tract infections (LRTI) are a leading global cause of mortality in all age groups [1, 2]. For example, in the Unites States of America, acute LRTI are associated with a greater morbidity and mortality than any other infection (https://www.cdc.gov/nchs/fastats/leading-causes-of-death.htm). Further, in Europe, pneumonia is associated with approximately 230,000 deaths per year [3]. However, the accurate differentiation of a bacterial infection, viral infection or no infection in cases of lower respiratory tract complaints (useful in deciding if a clinician should prescribe antibiotics or not to patients), is still difficult. Expert guidelines to direct this decision-making process have been available for several years [4, 5] with many algorithm-based guidelines incorporating the popular biomarker C-reactive protein (CRP) and/or procalcitonin (PCT) for the presumed diagnosis of an infection [6–9]. Further, biomarkers continue to be described as being potentially useful in differentiating between bacterial versus viral respiratory infections/sepsis. These include for example: TNF-related apoptosis-inducing ligand (TRAIL) [10], Interferon gamma induced protein-10 (IP-10) [11], Myxoma resistance protein (MxA1) [12, 13] and Lipocalin-2 (Lcn2) [14]. Diagnostic algorithms incorporating such biomarkers may help guide targeted antibiotic prescribing for bacterial LRTI, helping reduce (long term) costs and possible unnecessary side-effects in cases of viral LRTI, while helping reduce the increasing global epidemic of antibiotic resistance [15, 16].

Interestingly, research has also implicated the respiratory tract microbiota in the etiology of LRTI, with a particular emphasis on the nasopharyngeal microbiota [17, 18], although the actual site of sampling may be instrumental in the success of such approaches [19]. However, with respect to practical considerations (including the performance of large clinical trials), taking a nasal swab tends to be more convenient for nurses and doctors, and more comfortable/acceptable for patients than taking a nasopharyngeal swab, nose/nasopharyngeal washing, tracheal aspirate or bronchoalveolar lavage [20].

The development of rapid sequencing techniques has facilitated the prospect of (rapid) microbiota profiling as a diagnostic parameter in infectious diseases to enhance diagnostic algorithms based on a patient’s personalized clinical data. Therefore, in this publication, we investigated the added value of nasal cavity microbiota profiling in increasing the predictive power of diagnostic algorithms for classifying bacterial versus viral infection in children and adults with LRTI. The basis for this research was the EU-funded FP7 TAILORED-Treatment study (Grant ID 602860), which was charged with establishing a multi-omics approach to aid effective antibiotic prescribing in lower respiratory tract infections. After rigorous testing, we observed that nasal cavity microbiota correlate with the clinical variables and thus do not add significant value in the classifiers to differentiate bacterial from viral infections. Our prediction model with four easy-to-collect clinical variables greatly improves current diagnosis practice and will further facilitate personalized and accurate clinical decision-making for patients with LRTI.

## Materials and methods

### Ethics statement

The TAILORED-Treatment study is registered on ClinicalTrials.gov in January 2014 under the registration number NCT02025699 and was approved by the ethics committees in the participating countries. All procedures performed in studies involving human participants were in accordance with the ethical standards of the institutional standards of the institutional and/or national research committee and with the 1964 Helsinki declaration and its later amendments or comparable ethical standards. Patients or their legal representatives (parents or guardians) received explanations about the study protocol and signed the study informed consent forms. Informed consent was obtained from all individual participants included in the study.

Specifically, ethical approval was obtained from the Medical Ethical Committee of UMCU (14–104) and the Institutional Review Boards of Hillel Yaffe Medical Centre (HYMC-0108-13 and HYMC-0107-13), Bnai Zion Medical Centre (BNZ-0107-14 and BNZ-0011-14) and Hadassah University Medical Centre (HMO-0007-14 and HMO-0006-14).

### Patient cohort and study design

Patients were recruited for the EU FP7-funded ‘TAILORED-Treatment’ study, which ran from April 2014 to September 2016 [21]. During this period, a total of 1,261 pediatric and adult patients aged 1 month and older with suspected LRTI and/or sepsis were recruited at 7 participating emergency departments and hospital wards of Dutch and Israeli medical centers. From these patients a total of 516 nasal cavity microbiota profiles met the quality criteria of the study, using 16S rRNA gene sequencing quality criteria of >950 16S rRNA molecules DNA/μl and a sequencing depth of >1000 reads per sample. One hundred and sixty four of these 516 profiles were subsequently removed from the study as the relevant patients did not match our expert panel criteria for the clinical definition of bacterial or viral infection, i.e. the patients were classified as ‘non-infectious’ or ‘undetermined’. Suspected mixed viral and bacterial infections were labelled as bacterial infections, as they often elicit similar patient management—in LRTI most therapy of patients is prescribed based on infection with bacterial not viral pathogens. An additional 59 patients were also excluded from further analysis due to missing CRP data (CRP is already used in clinical practice as an important predictive variable of inflammation/infection).

**Fig 1:**
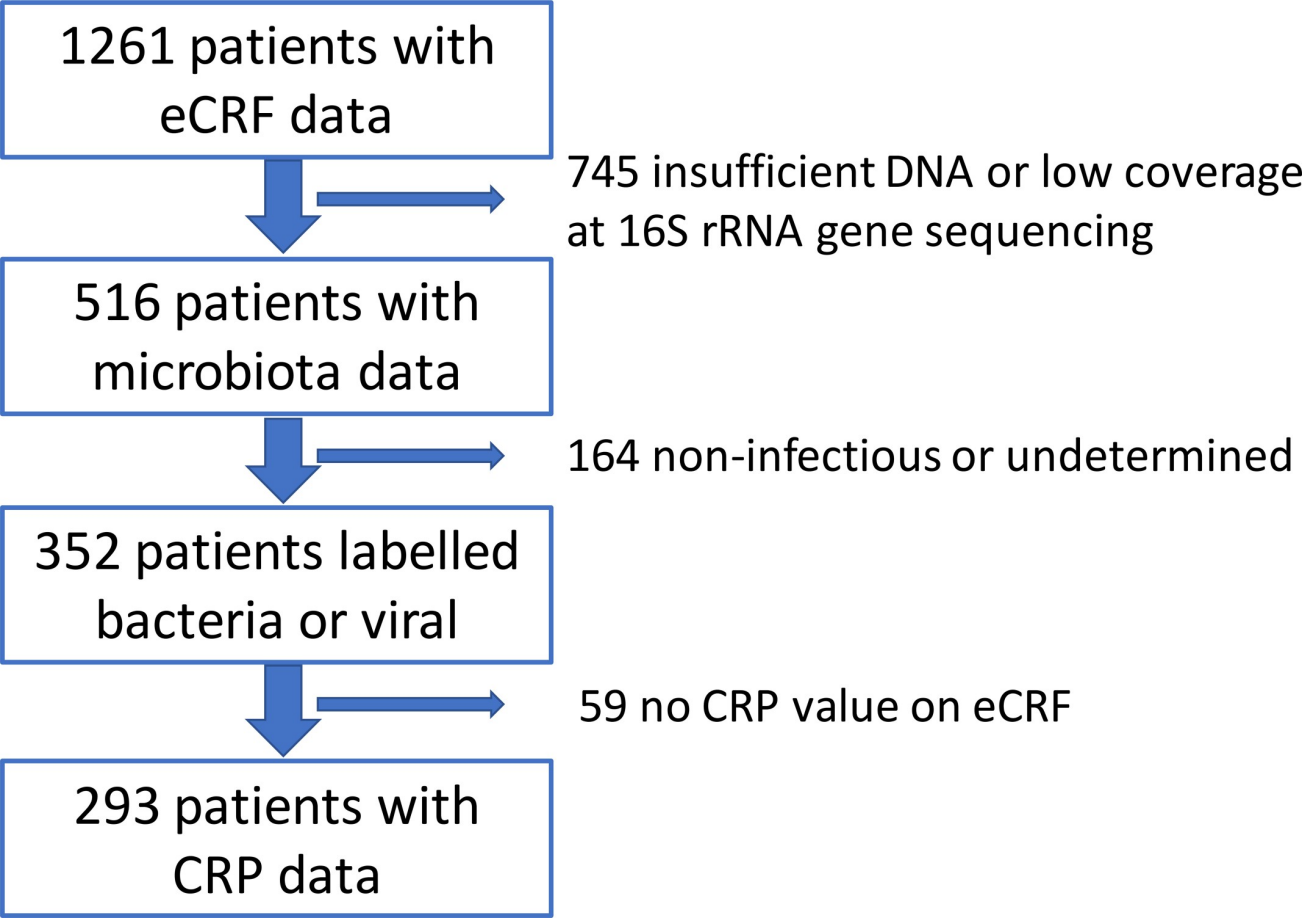
Patient filtering. LRTI patients from TAILORED-Treatment study underwent several filtering steps before they entered the classifier development stage. The eCRF data utilized in this publication was obtained from the following patient cohort [21]. eCRF: electronic Case Report Forms. CRP: C-reactive protein.

**Fig 2:**
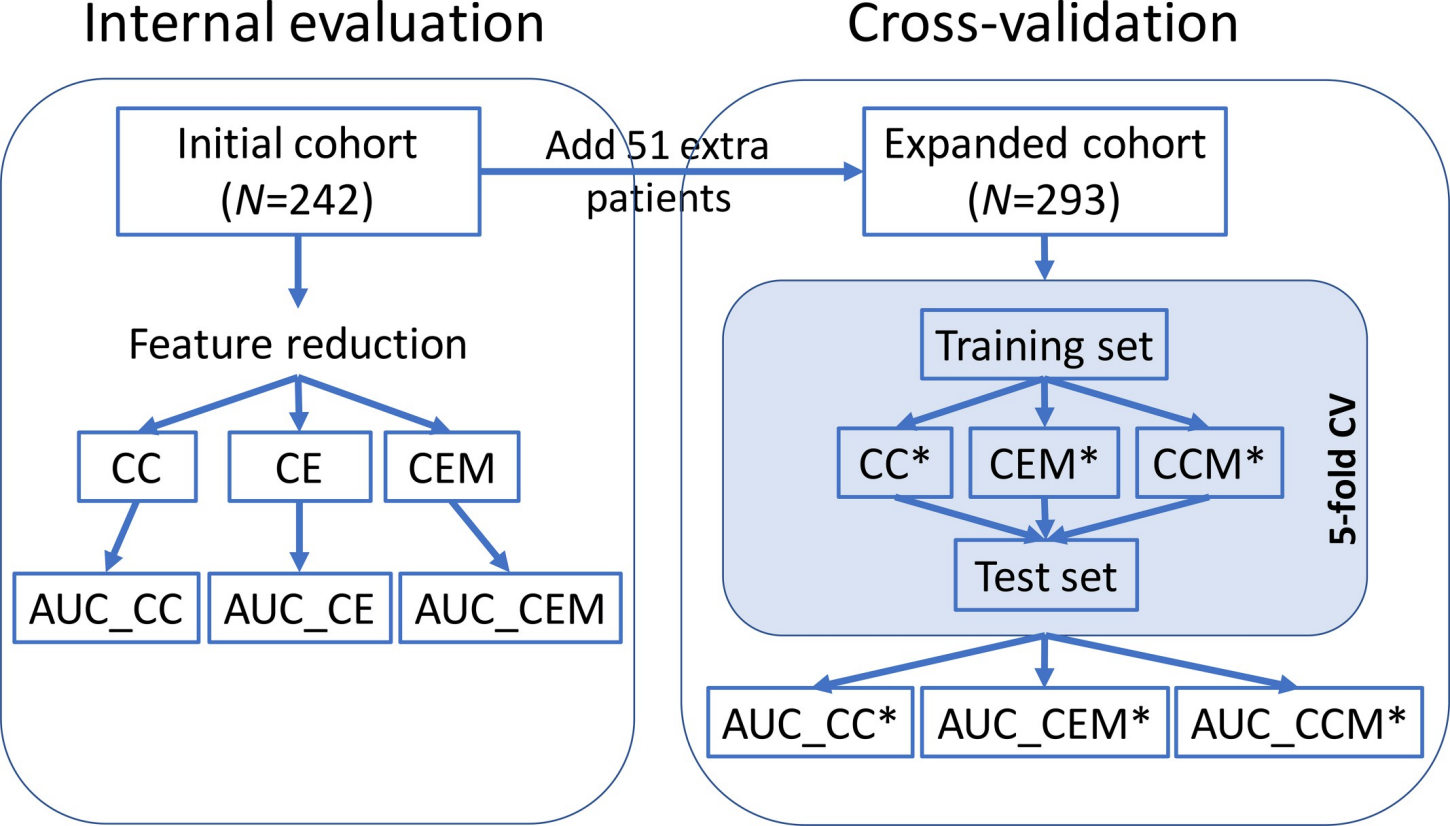
Study overview. A cohort of 242 patients were included in the internal evaluation phase according to the date of recruitment to the TAILORED-Treatment study. This cohort was used to compare the prediction performances of the classifiers using eCRF variables alone, as well as classifiers using both eCRF and microbiota variables (Internal evaluation phase). In the expanded cohort (51 extra patients), 5-fold cross-validation (CV) analysis was conducted to evaluate the contribution of eCRF and microbiota variables to prediction performance (Cross-validation phase). **CC**: **C**lassifier using **C**RP only in the initial cohort. **CE**: **C**lassifiers using two or more **e**CRF variables (incl. CRP) in the initial cohort. **CEM**: **C**lassifiers using all input **e**CRF variables (incl. CRP) and at least one **m**icrobiota in the initial cohort. CC*: **C**lassifier using **C**RP only in the 5-fold CV of the expanded cohort. CEM*: **C**lassifiers using two or more variables (regardless **e**CRF or **m**icrobiota) in the 5-fold CV of the expanded cohort. CCM*: **C**lassifiers using **C**RP and all input **m**icrobiota variables in the 5-fold CV of the expanded cohort. AUC: Area Under the ROC Curve.

Ultimately, this meant that machine learning classification analysis was performed on a total cohort of 293 patients (**Fig 1** and **S1 Table** for patient characteristics). By following the TAILORED-Treatment study protocol [21], a subset of 242 patients that were recruited during the discovery phase were included in the initial cohort. They were used to reduce uninformative variables and investigate whether adding microbiota data on top of clinical variables on the electronic Case Report Forms (eCRFs) helped to achieve better prediction of ‘bacterial infection’ versus ‘viral infection’ at patient presentation. Subsequently, the other 51 patients that were recruited later were added to the initial cohort in order to evaluate the two types of variables simultaneously (i.e. clinical and microbiota) in 5-fold cross-validation setting (**Fig 2**) to evaluate the generalization of the classification performance and to build the final classifier.

### Expert panel

A recently published reference standard using an expert panel protocol was used to assign TAILORED-Treatment study patients to either ‘bacterial infection’ or ‘viral infection’ class labels [16, 21]. The TAILORED-Treatment expert panel comprised 3 experienced physicians who were provided with all available clinical and laboratory information as listed in the electronic case record forms, including 28-day follow-up evaluation data. Each expert was blinded to the research results and to the labels of his/her peers on the expert panel. A final diagnosis was determined based on the consensus agreement among all three experts. Suspected mixed viral and bacterial infections were labelled as bacterial infections, as they often elicit similar patient management—in LRTI most therapy of patients is prescribed based on infection with bacterial not viral pathogens. Therefore, including ‘mixed’ infections in ‘bacterial infection’ will include all patients that may eventually require antibiotic therapy. Further, mixed viral and bacterial infections may actually lead to more serious disease and consequently bear more chance of antibiotic prescribing [22–24].

### Clinical eCRF data

Clinical eCRF data were generated using standard laboratory methods according to the published TAILORED-Treatment clinical trial protocol [21]. All clinical information as listed in the eCRFs was used by the expert panel to determine the etiology of infection. However, not all eCRF variables were actually included in the current prediction modelling as some of these variables were not available at patient presentation, and thereby not applicable for clinical diagnosis at patient presentation. In this respect, 195 eCRF variables were selected to be used in the analysis (**S2 Table**). After conversion of the categorical variables into Boolean data using one hot encoding, a total of 1,624 eCRF variables remained. This dataset was then cleaned to correct for input errors, to unify metrics and to merge variables within the same category. Variables only available for ≤2 patients were also removed, resulting in 58 numeric variables and 235 categorical variables available for further analysis.

In the internal evaluation phase, we performed feature reduction based on 242 patients in the initial TAILORED-Treatment cohort by testing the correlation between these 293 variables and the class labels (i.e. ‘bacterial infection’ or ‘viral infection’). More specifically, we tested the categorical variables by Fisher’s Exact test using R [25] function *fisher*.*test*. The resulting *p*-values were subject to Benjamini–Hochberg procedure to control for the False Discovery Rate (FDR). Variables having an adjusted *p*-value lower than 0.05 were retained for further classification modelling. For the numeric variables, we applied both parametric *t*-test and nonparametric Mann-Whitney *U* test. Twelve numeric variables generated significant results with *p*<0.05 in both tests. Among these, 4 redundant variables with a Pearson’s *r*>0.7 and *p*<0.05 were removed.

After feature reduction, 21 out of 235 categorical eCRF variables and 8 out of 58 numeric eCRF variables were identified that differed significantly between the ‘bacterial infection’ and ‘viral infection’ patients in the initial cohort. They served as the clinical input variables for the classifiers. All clinical data used for the prediction model can be found in **S3 Table**.

### 16S rRNA gene sequencing and pre-processing

Nasal cavity swab samples were collected from 1261 patients using e-swabs (Copan, USA) containing 1mL of liquid Amies medium and stored at -80°C for subsequent 16S rRNA gene sequencing analysis. DNA was extracted from all nasal cavity swab samples, using a phenol/bead-beating protocol combined with the AGOWA mag Mini DNA Isolation Kit (LGC) as described previously [26]. In addition, DNA from elution buffer BL (LGC) was extracted as negative extraction control samples at the same time to assess the composition of contaminating bacterial DNA in the experimental methodologies. 16S rRNA gene amplicon library preparation was performed as previously published [27, 28]. The hypervariable V5 and V6 regions (276 bp) of the 16S rRNA gene were amplified using the 785F (5′-GGA TTA GAT ACC CBR GTA GTC-3′) and 1061R (5′-TCA CGR CAC 20 GAG CTG ACG AC-3′) primers [27] and dual indexing [28]. Amplicons were generated in 30 cycli using the FastStart High Fidelity System (Roche), normalized using the SequalPrep Normalization Plate kit (Thermo Fischer Scientific) and pooled in batches of approximately 250 samples. Pools were purified prior to sequencing using the Agencourt AMPure XP (Beckman Coulter Life Science, Indianapolis, IN) and the amplicon size and quantity of the pools were assessed on the LabChip GX (PerkinElmer Inc., Groningen, The Netherlands). The PhiX Control v3 library (Illumina Inc., San Diego, CA) was combined (~10%) with the pooled amplicon libraries and each pool was sequenced on an Illumina MiSeq sequencer 2 (MiSeq Reagent Kit v3, 2 x 300 bp).

Bidirectional sequencing of the 16S rRNA gene amplicon libraries was performed using the Illumina MiSeq platform, with FASTQ-formatted sequences being extracted from the machine and further processed using a Galaxy mothur toolset [29] based on the standard mothur bioinformatics pipeline [30]. For those samples with more than 5000 reads, we randomly subsampled 5000 reads per sample which went further into the pipeline. Forward and reverse FASTQ-formatted sequence files were merged using the *make*.*contigs* command. Unique sequences were aligned against the SILVA [31] reference alignment release 123, where the reference sequences were trimmed to only include the V5-V6 region of the 16S rRNA gene using the *pcr*.*seqs* command. To filter out sequencing errors, we applied pre-clustering using the *pre*.*cluster* command allowing for up to two differences between sequences. Potential chimeric sequences were removed using UCHIME [32]. The remaining sequences were assigned to taxonomy using the *classify*.*seqs* command based on the customized SILVA alignment release 123. Sequences were then clustered into operational taxonomic units (OTUs) at 97% similarity using the *dist*.*seq* and the *cluster* commands. Finally, each OTU was assigned to a consensus taxonomy using the *classify*.*otu* command. For quality reasons, only samples generating >950 16S rRNA gene molecules/μl and >1000 reads were included in further analysis. Additional filtering to reduce noise involved removing OTUs with 2 or fewer reads, since they were very likely to be sequence artifacts e.g. chimeras. We calculated the relative abundance for each OTU, by dividing the OTU count number by the total count per sample. OTUs were then summarized into 240 genera for further analysis. Next, to avoid overfitting the model of genera that may not be representative in the general LRTI patient population, the relative genus-level abundance data was transformed and filtered according to recommendations in Rhea [33] in two steps: 1) All relative abundances in any sample below 0.5% were considered as absent (i.e. missing value) and 2) for prevalence-based filtering—if a genus was present in <30% of samples in both ‘bacterial infection’ and ‘viral infection’ classes, then the genus was considered ‘too sparse’ and was not included for further analysis.

### Classification modelling

Several classifiers were built in the initial cohort as well as in the expanded cohort and their performances were evaluated. Since CRP is widely used in current clinical practice to detect inflammation/infection, the classification performance using only CRP (i.e. CC and CC* in **Fig 2**) serves as the benchmark. Besides CRP, we would like to investigate the prediction value of readily available clinical variables (i.e. CE in **Fig 2**) and the added value of microbiota (i.e. CEM, CEM* and CCM* in **Fig 2**). The initial cohort was dedicated to reduce uninformative variables and investigate whether adding microbiota data on top of clinical variables (i.e. CEM vs. CE) helped to achieve better prediction of ‘bacterial infection’ versus ‘viral infection’ at patient presentation. The expanded cohort not only contained more patient samples, but also was used to evaluate clinical and microbiota variables simultaneously for a fair comparison. That is, instead of adding the microbiota variables after the clinical ones as in the initial cohort (i.e. CEM), here all input variables entered the modelling without imposed order to rank their importance for prediction (i.e. CEM*).

Given the nature of our dataset (a mix of categorical and numeric variables with missing data), Breiman’s Random Forests classification method [34] was chosen and implemented using R package *randomForestSRC* version 2.11.0. The advantage of using Random Forest as the classification model in this case is three-fold: 1) It can handle both categorical data (some of the clinical variables) and numeric data (microbiota and some of the clinical variables); 2) It is a tree-based model and hence does not require feature scaling; 3) It can handle missing values by using inference or imputation. Random Forests are ensembles of decision trees which vote for the most popular class. When growing these ensembles, bagging (i.e. bootstrap aggregating) [35], is used in tandem with random feature selection. To grow each decision tree in the training phase, a new sub-training set is drawn with replacement from the original training dataset while about one-third of the samples are left out (i.e. out-of-bag). Then a tree is grown on the new sub-training set using random feature selection. The out-of-bag samples are used to get a running unbiased estimate of the classification error as they are excluded in the sub-training set for that particular tree. Therefore, this method can provide an unbiased error rate estimate from the out-of-bag error rate even internally without the need for cross-validation or a separate test set. In our implementation, R package *randomForestSRC* was deployed using *mtry* = NULL, *ntree* = 500, and *na*.*action* = "na.impute". Missing data were imputed using in-bag non-missing data only, via a modification of the missing data algorithm of Ishwaran *et al*. [36].

## Results

### Nasal cavity microbiota

**Fig 3:**
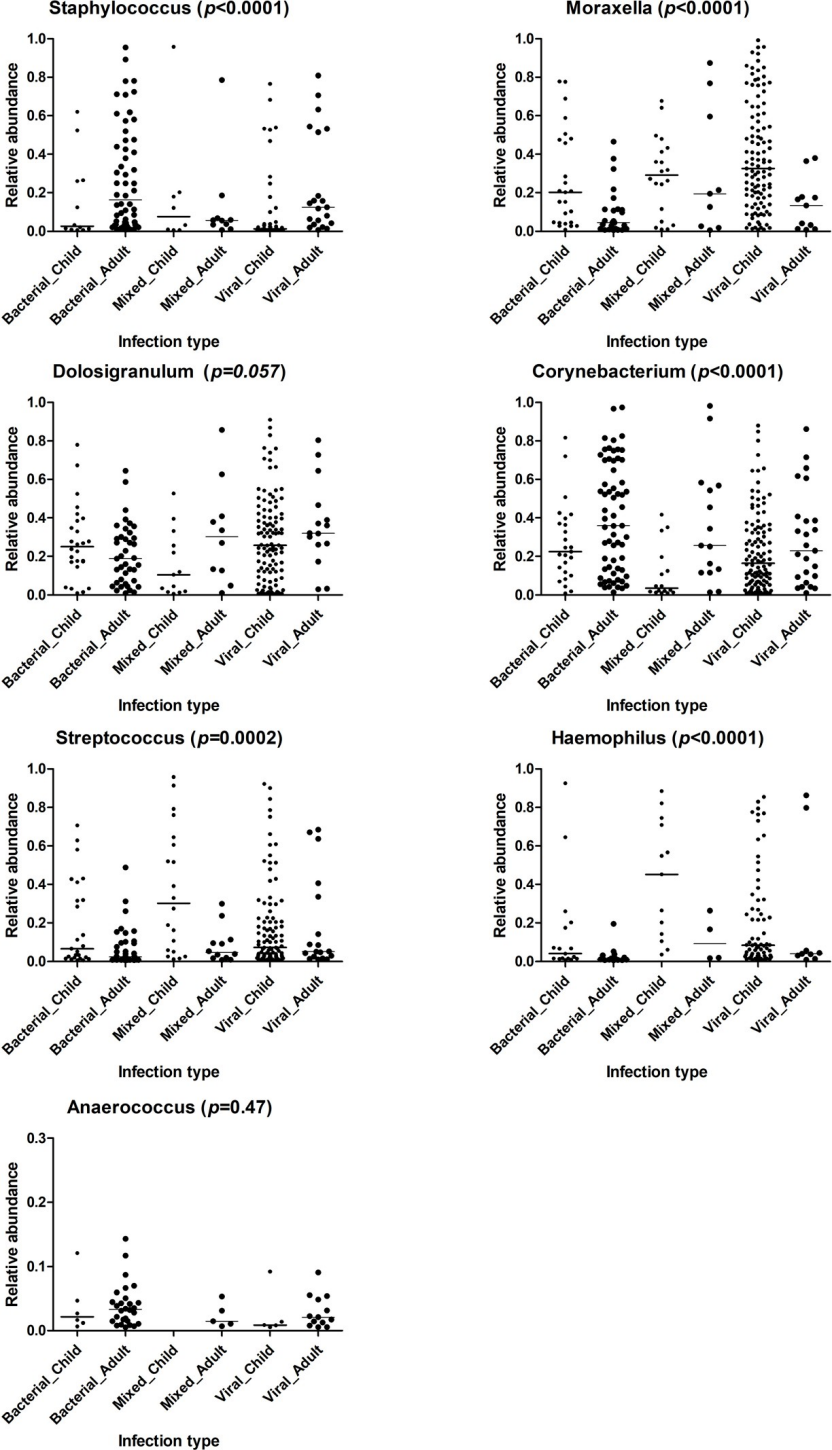
Relative abundance of seven most common bacterial genera related to age and infection origin of TAILORED-Treatment cohort. Kruskal-Wallis test was performed to calculate the *p*-values. Horizontal bars represent the median values.

The average sequence coverage per sample was 88,461. Pre-clustering, chimera removal and operational taxonomic units (OTU) clustering at a similarity of 97% identified a total of 2,838 OTUs. After filtering out OTUs with 2 or fewer reads, the relative abundance were calculated for the remaining OTUs which were then summarized into 240 genera for further analysis. The genus-level relative abundance data was transformed and filtered according to recommendations in Rhea [33] (see Materials and Methods). Seven genera remained after Rhea filtering and served as the input for the classification modelling of the microbiota variables. These genera were: *Staphylococcus*, *Moraxella*, *Dolosigranulum*, *Corynebacterium*, *Streptococcus*, *Haemophilus*, and *Anaerococcus*, which have already been associated with the nasal microbiota in many publications, including in infants, children and adults [37–40]. **Fig 3** shows the relative abundance of these seven genera in relation to age and infection origin of the TAILORED-Treatment cohort. Although suspected mixed viral and bacterial infections were labelled as bacterial infections, we list them separately in this figure in order to highlight the differences between “mixed” (bacterial and viral) and “bacterial” infections (see Discussion). All microbiota data used for the prediction model can be found in **S3 Table**.

### Classification performance & variable importance

In total, 29 clinical variables and 7 microbiota variables entered the classifier modelling phase (**S1 Fig**). Notably, more than 50% of data was missing for 6 of these 36 variables. In both phases, the performance of the models was assessed by calculating the area under the receiver operating characteristic curve (AUC) for overall performance and the percentage of correctly predicted cases.

In the internal evaluation phase, 242 patients were included to compare the prediction performances of classifiers using eCRF variables alone and classifiers using both eCRF and microbiota variables to assess whether adding microbiota on top of clinical data helped to distinguish bacterial infection from viral infection (**Fig 2**). More specifically, we first ranked the 29 input eCRF variables and 7 nasal cavity microbiota variables separately by function *vimp* in the initial cohort [34] (**S1 Fig**), then the eCRF variables were included into the Random Forests model incrementally according to their importance ranking and subsequently the ranked nasal cavity microbiota variables. Each time 1000 forests were grown and the best forest was kept. This was iterated 10 times to evaluate the robustness of the model.

**Fig 4:**
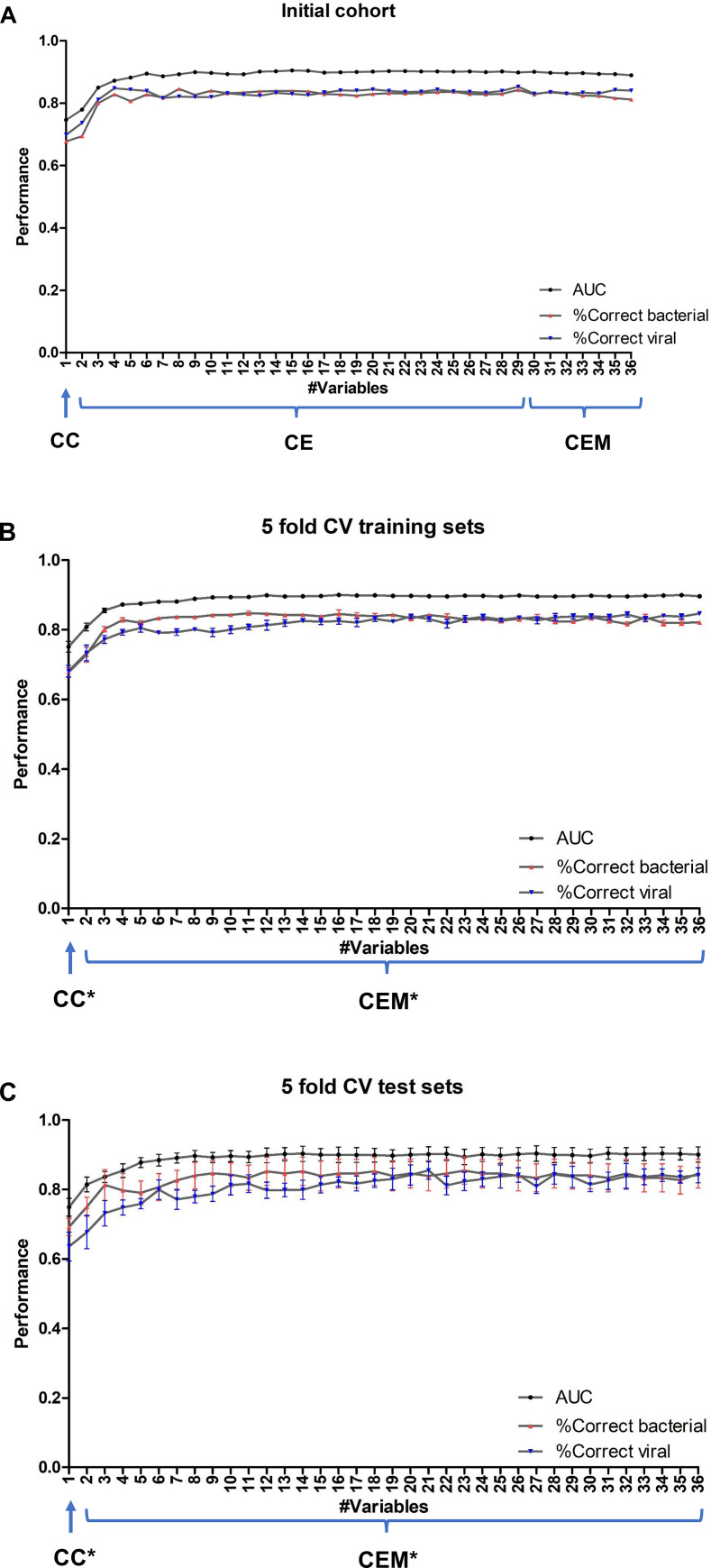
Performance of the classifiers. Classifier performance in **A**) the initial cohort, **B**) 5-fold cross-validation training sets in the expanded cohort, and **C**) 5-fold cross-validation test sets in the expanded cohort. X-axis shows the number of variables included in the classifier. The lines represent the mean of AUC, the accuracy of class ‘bacterial infection’, and the accuracy of class ‘viral infection’, respectively. The bars represent the standard error of the mean (SEM). In the initial cohort (Panel A), first eCRF variables were ranked separately and included in the classifier incrementally, followed by ranked microbiota variables. The ranking was based on their variable importance calculated by function *vimp* in the initial cohort. In the cross-validation (Panels B-C), the ranking of all eCRF and microbiota variables was calculated simultaneously based on the training set in the particular split and averaged across five splits. **CC**: Classifier using CRP only in the initial cohort. **CE**: Classifiers using two or more eCRF variables (incl. CRP) in the initial cohort. **CEM**: Classifiers using all input eCRF variables (incl. CRP) and at least one nasal cavity microbiota variable in the initial cohort. CC*: Classifier using only CRP in the 5-fold cross-validation of the expanded cohort. CEM*: Classifiers using two or more variables (regardless of eCRF or nasal cavity microbiota origin) in the 5-fold cross-validation of the expanded cohort. AUC: Area Under the ROC Curve. CV: cross-validation. SEM: standard error of the mean.

The resulting AUC and accuracy per class are shown in **Fig 4A** and **S4 Table**, which are out-of-bag error rates calculated by *randomForestSRC*. More specifically, the classifier using the best eCRF variable CRP alone (“CC”), generated an average AUC of 0.75 in the 10 times repetitions and accuracies of 68% for bacterial infection and 70% for viral infection cases. Further, the AUC and accuracies for the prediction of both classes were greatly improved, and remained stable, after adding the first few (non-microbiota) clinical variables into classifiers “CE”. The average AUC of CE ranges from 0.78 to 0.91 when using 2 to 29 eCRF variables, with the maximum AUC being reached when using the top ranked 15 eCRF variables. However, adding nasal cavity microbiota variables on top of these 29 eCRF variables into classifiers “CEM” did not increase the AUC or the accuracy of the classifier. The average AUC of CEM was between 0.89 and 0.90 using all 29 eCRF variables plus 1 to 7 microbiota genera (**S4 Table**).

In the expanded cohort with 51 extra patients, 5-fold cross-validation was conducted to evaluate the contribution of the eCRF and microbiota variables to the prediction performance. The total cohort of 293 patients was randomly split into 5 subsets, or so called ‘folds’. Each time one fold containing 58 or 59 patients was taken as the test set and the remaining 4 folders formed the training set. In each round, the test fold was withheld in the training step and used to assess the prediction accuracy on “unseen” data in the test fold. Eventually all samples in the dataset were evaluated exactly once to obtain the overall performance of the models. Since the two classes were unbalanced, the majority class label ‘viral infection’ was randomly subsampled to reach equal prior probability as class ‘bacterial infection’ in each training set. Unlike the internal evaluation phase where the eCRF and microbiota variables were ranked separately and added sequentially (i.e. first the eCRF variables and then the microbiota ones), here all variables were ranked simultaneously based on the training set at hand using function *vimp* and added into the model accordingly.

In the 5-fold CV, classification performance was assessed on both training set (using ‘out-of-bag’ predictions) and test set at hand. We then summarized the performance metrics by taking the mean and standard error of the mean. The resulting AUC graphs and accuracy per class of the classifier using CRP alone (“CC*”) and classifiers using two or more variables (“CEM*”) are shown in **Fig 4B and 4C** and **S4 Table**. In both the training sets and test sets of 5-fold cross-validation, the classifier using CRP alone generated an average AUC of 0.75 which is the same as in the internal evaluation phase. The average accuracies of bacterial and viral classes in the test sets were 69% and 64%, respectively. Both the AUC and accuracy per class were consistently improved after adding the first few variables and remained stable upon the addition of subsequent variables. More specifically, adding 3 more clinical variables, including ‘absolute neutrophil count’ (ANC), ‘consolidation on X-ray’, and ‘age group’ on top of CRP significantly improved the prediction towards an average AUC of 0.85 in the 5-fold cross-validation test sets. The distribution of the four most predictive variables can be found in **S2 Fig**. The highest average AUC in the 5 training sets was reached using the top 16 variables (**S5 Table**), and the average performance using these variables in the 5 test sets was an AUC of 0.90, with 85% of bacterial infection and 82% of viral infection being correctly predicted. Of note, 3 genera were present in the top 16 variables: *Staphylococcus* (rank #11), *Moraxella* (rank #13) and *Streptococcus* (rank #14).

We also conducted 5-fold cross-validation to investigate the added value of adding nasal cavity microbiota variables to the best eCRF predictor, i.e. CRP. That is, classifier CCM* was built using CRP plus all 7 microbiota genera variables as the input variables. Compared to CRP alone, adding nasal cavity microbiota variables showed a slight improvement in AUC (from 0.75 to 0.81), which held for both training sets and test sets (**S4 Table**). The percentage of correctly predicted ‘viral infection’ patients in the test sets was pronouncedly improved (from 63% to 80%) after adding 7 nasal cavity microbiota variables to CRP, whereas the percentage of correctly predicted cases of ‘bacterial infection’ patients was only slightly improved (from 69% to 74%).

The use of the Random Forests model meant that unbiased error rate estimate could be derived internally from the out-of-bag samples without the need for cross-validation or a separate test set, because the out-of-bag samples are excluded in the training set for the particular tree to obtain a running unbiased estimate of the classification error. Indeed, we observed very similar performance in our training set (using the out-of-bag samples) and test sets (using independent samples) as shown in **Fig 4B and 4C**. This demonstrates the robustness of our prediction model. Further, the standard error of mean (SEM) in the test sets was modest, suggesting that the sample size of the cohort used in this study was sufficient.

### Correlations between nasal cavity microbiota and clinical variables

Since we observed no improvement after adding nasal cavity microbiota variables into the classifiers when using all eCRF variables, we hypothesized that nasal cavity microbiota variables were probably correlated with the eCRF variables and thereby provided limited extra predictive value. Based on this hypothesis, we investigated the correlations between the 7 genera (nasal cavity microbiota) variables and 29 clinical (eCRF) variables in the initial cohort for each class separately. Spearman’s *rho* was calculated for numeric variables, and Mann-Whitney *U* test or Kruskal–Wallis test was performed to calculate the correlation between numeric variables and categorical variables. The results are shown in **S6 Table**, together with the correlations among the 7 genera. As we expected, all 7 nasal cavity microbiota variables were correlated with one or more clinical variables. In particular, *Corynebacterium* and *Streptococcus* genera were significantly correlated with the highest number of clinical variables (both 11), while *Dolosigranulum* and *Anaerococcus* were significantly correlated with the least number of clinical variables (1 and 2, respectively).

## Discussion

In this publication, we describe the development of a comprehensive machine learning algorithm for distinguishing ‘bacterial infection’ from ‘viral infection’ in a cohort comprising child and adult patients presenting with LRTI. The TAILORED-Treatment study methodology and cohort recruitment allowed an assessment to be made of easy-to-collect clinical variables together with nasal cavity microbiota variables for predicting ‘bacterial infection’ versus ‘viral infection’. After rigorous testing, we observed that nasal cavity microbiota correlate with the clinical variables and thus do not add significant value besides the clinical variables in the classifiers to differentiate bacterial from viral infections. Our findings indicate that 4 clinical variables, i.e. CRP, absolute neutrophil count (ANC), consolidation on X-ray and age group, gave an average AUC of 0.85 and that the addition of nasal cavity microbiota variables leads to a marginal improvement in the accuracy of the algorithm.

Since this marginal improvement may not be sufficient to affect the antibiotic prescribing decision of clinicians in patients presenting with LRTI, our study provides evidence of limited clinical value of using nasal microbiota data for infection diagnosis. Furthermore, we propose an accurate multi-parametric prediction model which takes the interactions between multi-factors into account and is applicable for both child and adult patients. Our prediction model with four easy-to-collect clinical variables greatly improves current diagnosis practice and will further facilitate personalized and accurate clinical decision-making for patients with LRTI.

The data presented in this publication was collected as part of a European-wide approach to combat ever increasing global antibiotic resistance. We expect to demonstrate our prediction model in a future cohort should it be available. Targeted antibiotic prescribing to patients suffering from bacterial, as opposed to viral, infections could potentially limit the amount of unnecessary antibiotics prescribed by clinicians (TAILORED-Treatment) [16]. Better targeted antibiotic treatment involving a reduction in the unnecessary prescription of antibiotics at local, national and international levels, will help limit the increasing prevalence of antibiotic resistance, as well as inhibit the development of new antibiotic resistances.

### The nasal cavity microbiota

The use of established 16S rRNA gene sequencing techniques has shown that the respiratory tract microbiota varies with age, with Proteobacteria (*Haemophilus*, *Neisseria* and *Moraxella*), and Firmicutes (*Strepotcoccus*, *Gemella*, *Dolosogranulum* and *Granulicatella*) being overrepresented in prepubertal children and Actinobacteria (*Corynebacteium*, *Propionibacterium* and *Turicella*) being overrepresented in adults [41]. The presence of potential bacterial pathogens in the respiratory microbiota have also been associated with LRTI. This variation usually involves colonization with *Streptococcus pneumoniae*, *Haemophilus influenzae* and *Moraxella catarrhalis* (three bacteria often associated with the development of upper respiratory tract infections in infants, including otitis media in young children) and *Staphylococcus aureus*, *Dolosigranulum* spp. or *Corynebacterium* spp. [42–44]. However, published results tend to be presented for either a mixture of both nasal and nasopharynx microbiota or only the nasopharyngeal microbiota [22, 45], while differences in the microbiota of nasal, nasopharyngeal and oropharyngeal microbiota and over time have been reported [41, 46]. Importantly, there are several physical reasons why the nasal cavity microbiota may vary from the nasopharyngeal microbiota including: 1) the presence of stiff coarse hairs (vibrissae) in the anterior nares; 2) epithelium type and 3) the cooler environment present compared to the nasopharynx [47]. Also, the sampling methodology used may affect the microbiota composition found from nasal/nasopharyngeal sites, with nasal washings not being ‘spatially specific’ [48]. In this respect, de Boeck *et al*. compared the healthy nose and nasopharynx microbiota, with the observation that both niches possessed a low overall species richness and uneven distribution and the nasopharynx was found to possess more pathogenic bacterial species than the nose [49]. A previous publication by Toivonen *et al*. indicated a role for the nasal microbiota in an increased rate of acute respiratory infection (ARI) in children up to 2 years of age [50]—note that the TAILORED-Treatment cohort involved child and adult participants combined. Interestingly, we also identified 3 out of the 5 microbiota genera (i.e. *Moraxella*, *Streptococcus*, *Dolosigranulum*, *Staphylococcus* and *Corynebacteriacea*) described by Toivonen as potential contributors to an enhanced ‘bacterial infection’ versus ‘viral infection’ diagnostic algorithm. However, our findings indicated that the addition of such microbiota variables only marginally increased the predictive power of our combined child and adult diagnostic algorithm. Luna *et al*. indicated that although the microbiota and the anterior nares and nasopharynx are distinct, there may be “considerable” overlap between microbiota genera from these two sites (*Haemophilus* and *Moraxella*, but not *Staphylococcus* genera), at least in infants hospitalized with bronchiolitis [20]. The authors suggested that nasal swabs are “effective sample types” and can be used “to detect microbial risk markers”. Again, the cohort characteristics of this publication were somewhat different (infants <1 year of age) as compared to the current publication and our study was not established to investigate bronchiolitis alone.

Finally, the nasal microbiota data used in our multi-parametric modelling was available at genus level due to limitations in the short-read sequencing technology used [51, 52]. As some microbiota genera found in the nose could contain pure commensal and potentially pathogenic species of bacteria, the utilisation of species level data, e.g. via long-read sequencing technologies, may generate increased accuracy in the development of nasal microbiota-based algorithms [53].

### CRP as biomarker

The most widely used biomarker for the detection of inflammation and guidance of clinical decision-making in primary, secondary and tertiary care is C-reactive protein (CRP). A recent meta-analysis and review concluded that the use of CRP-based algorithms “seems to reduce antibiotic treatment duration in neonates, as well as to decrease antibiotic treatment initiation in adult outpatients” [54]. This finding was also verified by Verbakel *et al*., who in a 2019 systemic review and analysis concluded that “performing a point-of-care CRP test in ambulatory care accompanied by clinical guidance on interpretation reduces the immediate antibiotic prescribing in both adults and children” [55]. It is therefore not surprising, and indeed reassuring, that CRP is one of the major clinical factors identified in the TAILORED-Treatment algorithm. However, that said, the use of CRP in helping antibiotic prescribing decision-making processes may not always be obvious and may depend on factors not directly associated with CRP measurement per se [56, 57]. Point-of-Care (POC) diagnostic devices now exist for the measurement of CRP in different clinical environments e.g. QuikRead go (Orion diagnostics) [58, 59]. Further, novel diagnostic algorithms continue to be developed in spite of the relative success of CRP as a biomarker in clinical decision-making, largely due to the different circumstances in which CRP may be utilised and the ‘grey zone’ that exists in the 20–100 mg/l range [42]. In this respect, rapid biomarker-based diagnostics are currently available, or are being developed, that may more accurately distinguish between bacterial and viral infections in cases of infection, by using multiple biomarkers. Some examples of these diagnostics include: 1) CRP and Myxovirus Resistance Protein 1 (MxA1)—FebriDx^®^ (RPS Inc., USA) [60]; 2) tumour necrosis factor-related apoptosis-inducing ligand (TRAIL), interferon gamma induced protein-10 (IP-10) and CRP—MeMed Key™ (MeMed BV, Israel) [61] and 3) Cathepsin B (CTSB), Hexokinase 3 (HK3), Interferon alpha inducible protein 27 (IFI27)—HostDx Tests (Inflammatix Inc., USA) [62].

### Mixed infections

After 5-fold cross-validation, all 293 patients obtained their predicted class labels in the test sets. Subsequently, we investigated the prediction of the classifier using all 36 variables. More specifically, 22 out of 161 ‘viral’ patients were misclassified as ‘bacterial’, while 24 out of 132 ‘bacterial’ patients were misclassified as ‘viral’. When we looked into these 24 misclassified ‘bacterial’ cases, we found that 15 of misclassified patients were actually associated with ‘mixed’ infection i.e. the expert panel had indicated that these patients were likely suffering from a viral and bacterial co-infection. Further, Fisher’s exact test showed that ‘mixed’ patients were significantly more likely to be misclassified (*p* = 0.0001). We can also see the differences of the microbiota values between “mixed” and “bacterial” patients in **Fig 3**. In this study we treat these co-infected patients as ‘bacterial infection’ since they also are likely to require antibiotic treatment. However, inclusion of ‘mixed’ infections in the ‘bacterial’ infection category inevitably decreased the accuracy of predicting the ‘bacterial infection’ class. We tested this using the classifier with all 36 variables: when the ‘mixed’ patients were removed from the cohort, the classifier’s AUC was increased from originally 0.90 to 0.94, and the accuracy of predicting the ‘bacterial infection’ class was improved to 87% while the accuracy of ‘viral infection’ class remained almost unchanged.

The algorithm developed in this study was designed to help clinicians decide on a bacterial versus viral infection at patient presentation. However, in real life situations, we cannot always be sure that these ‘mixed’ infections will be rapidly identified since clinicians do not have information immediately available on possible combinations of viral and bacterial infection. Consequently, the ‘bacterial infection’ class may include ‘mixed’ patients not identified as such at patient presentation. In this sense, the classification performance we report in this publication is an underestimation, with better algorithm performance being achieved if patients can be divided into strict ‘bacterial’ class at presentation where no viral infection is present. Of course, viral and bacterial infections often go hand in hand, so defining a strictly ‘bacterial’ class patient population may not be realistic.

## Supporting information

S1 Fig Input variables and missing data. The selected input variables for the classifiers and their respective missing data percentages. (A) 29 clinical eCRF variables and (B) 7 microbiota variables at genus level after Rhea transformation, i.e. all relative abundances in any sample below 0.5% were considered as absent. The variables are sorted by the importance calculated based on the initial cohort (see Methods). *: Numeric variables.
(TIF)

S2 Fig Distribution of the four most predictive variables in the expanded cohort of 293 patients. (A) C-reactive protein (CRP). (B) Absolute neutrophil count (ANC). (C) X-ray signs Consolidation. (D) Age group. In (A) and (B), the mean and standard error of mean are shown. In (C) and (D), the number of patients per category is shown. The patients with mixed infection are indicated separately although they were calculated as possessing ‘bacterial infection’ class labels in this study.
(ZIP)

S1 Table Clinical characteristics for the cohort of 293 patients, including (A) adult and (B) child age group. (A) Age group “Adult”. (B) Age group “Child” (<18 years old). For each characteristic, the (mean) value, percentage, and range are shown, together with a *p*-value by two-tailed *t*-test for numeric data or chi-square test for categorical data. *Excluding the missing values. ^**#**^By Fisher’s Exact test.
(XLSX)

S2 Table 195 clinical eCRF variables as the input to the analysis. (XLSX)

S3 Table All clinical and microbiota data used for the prediction model. (XLSX)

S4 Table Summary of the performance of classifiers. CC: Classifier using CRP only in the initial cohort. CE: Classifiers using two or more eCRF variables (incl. CRP) in the initial cohort. CEM: Classifiers using all input eCRF variables (incl. CRP) and at least one nasal cavity microbiota variable in the initial cohort. CC*: Classifier using only CRP in the 5-fold cross-validation of the expanded cohort. CEM*: Classifiers using two or more variables (regardless of eCRF or nasal cavity microbiota origin) in the 5-fold cross-validation of the expanded cohort. CCM*: Classifier using CRP plus all 7 microbiota genera variables in the 5-fold cross-validation of the expanded cohort. AUC: Area Under the ROC Curve. SEM: standard error of the mean.
(XLSX)

S5 Table Top sixteen predictive variables in the 5-fold cross-validation training sets. Given each training set in the 5-fold cross-validation of the expanded cohort, all input variables were ranked by their individual variable importance [34]. The overall ranking in the five training sets is shown here.
(XLSX)

S6 Table Correlations between microbiota and eCRF variables in the initial cohort. For the significant correlations between the 7 microbiota and 21 categorical eCRF variables (upper part of the table), Mann-Whitney *U* test or Kruskal–Wallis two-tailed test *p*-values are shown. For the significant correlations between the 7 microbiota and numeric variables (incl. 8 numeric eCRF variables and the other microbiota variables, lower part of the table), Spearman’s *rho* values are shown. These results were generated per class: those in class ‘bacterial infection’ have a superscript ‘b’ and those in class ‘viral infection’ have a superscript ‘v’. Results with *p*-val<0.05 were considered significant.
(XLSX)

S7 Table Metadata for the 16S RNA sequencing dataset. (XLSX)

## List of abbreviations

ANC: Absolute neutrophil count
ARI: Acute respiratory infection
AUC: Receiver operating characteristic curve
CRP: C-Reactive protein
CTSB: Cathepsin B
eCRF: Electronic case report form
FDR: False discovery rate
HK3: Hexokinase 3
HRV: Human rhinovirus
IFI27: Interferon alpha inducible protein 27
IP-10: Interferon gamma induced protein-10
Lcn2: Lipocalin-2
LRTI: Lower respiratory tract infections
MxA1: Myxoma resistance protein
OTU: Operational taxonomic units
PCT: Procalicitonin
POC: Point-of-Care
SEM: Standard error of mean
TRAIL: TNF-related apoptosis-inducing ligand
