# Supplementary figures and images for: The diagnostic value of nasal microbiota and clinical parameters in a multi-parametric prediction model to differentiate bacterial versus viral infections in lower respiratory tract infections
Source: PLoS One. 2022 Apr 18;17(4):e0267140. doi: 10.1371/journal.pone.0267140 (PMC9015155; doi:10.1371/journal.pone.0267140)

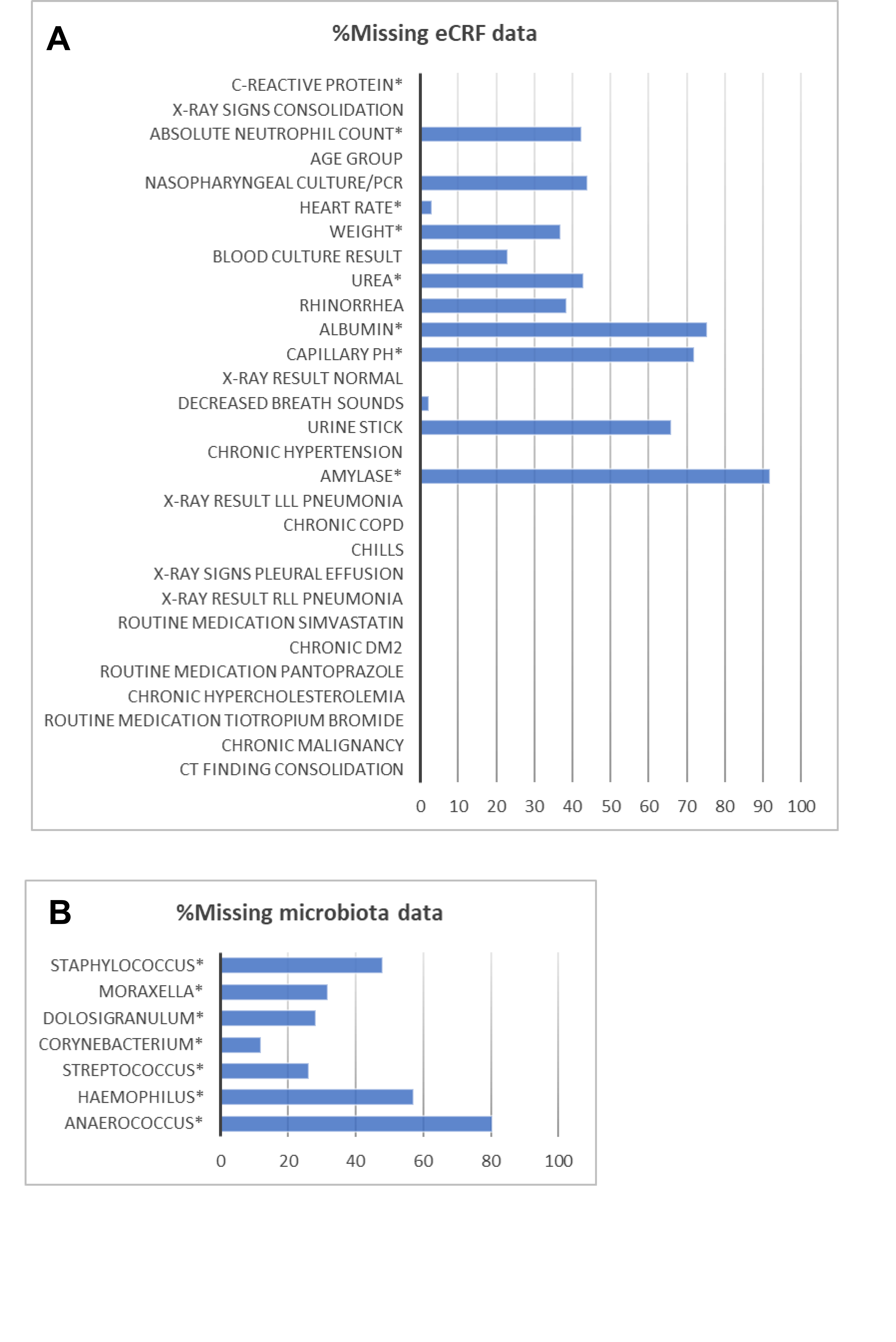

Supplement: S1 Fig — The selected input variables for the classifiers and their respective missing data percentages. (A) 29 clinical eCRF variables and (B) 7 microbiota variables at genus level after Rhea transformation, i.e. all relative abundances in any sample below 0.5% were considered as absent. The variables are sorted by the importance calculated based on the initial cohort (see Methods). *: Numeric variables. (TIF) [file pone.0267140.s001.tif]

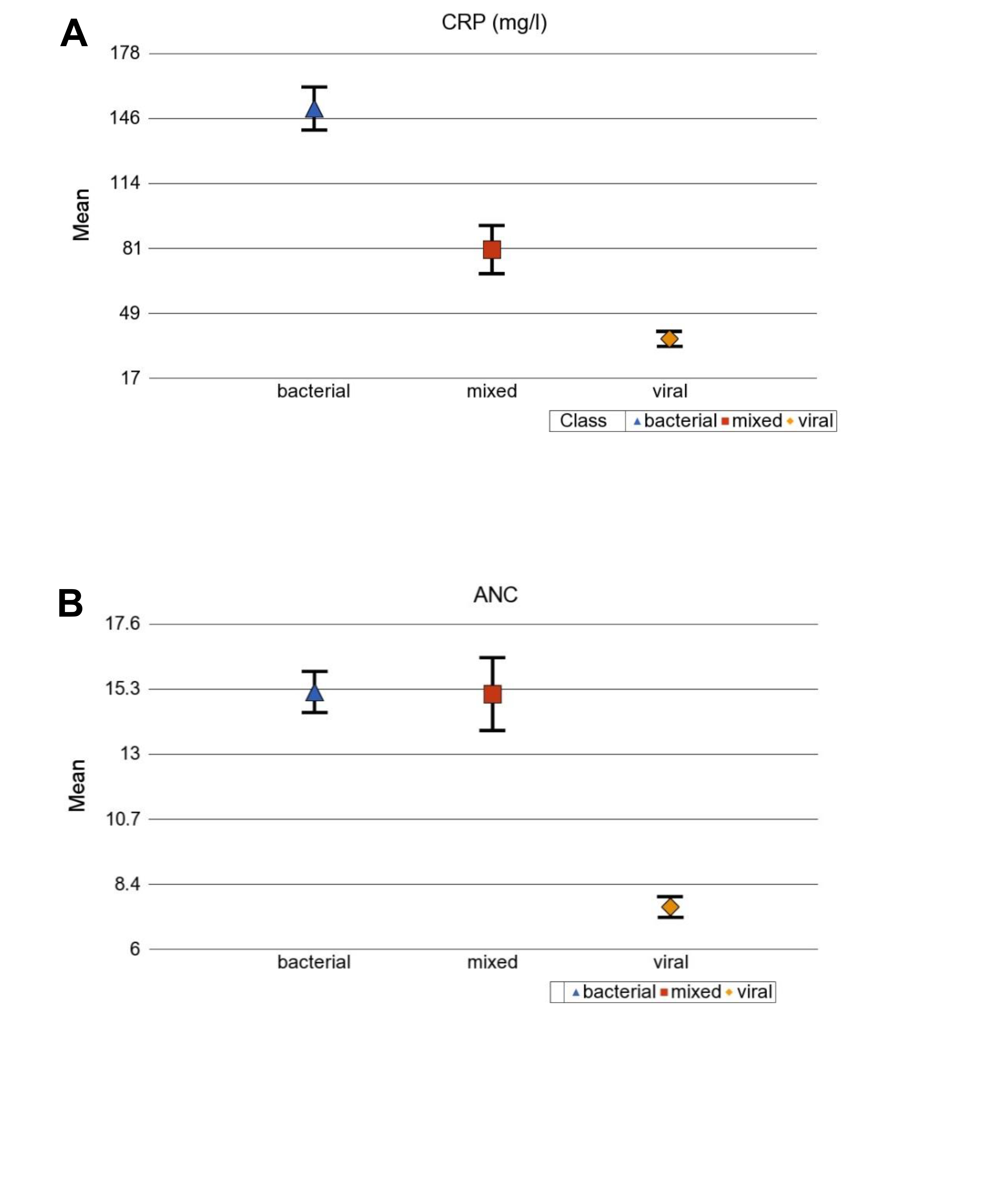

Supplement: S2 Fig — (A) C-reactive protein (CRP). (B) Absolute neutrophil count (ANC). (C) X-ray signs Consolidation. (D) Age group. In (A) and (B), the mean and standard error of mean are shown. In (C) and (D), the number of patients per category is shown. The patients with mixed infection are indicated separately although they were calculated as possessing ‘bacterial infection’ class labels in this study. (ZIP) [file pone.0267140.s002.zip › S2_Fig_A-B.tif]

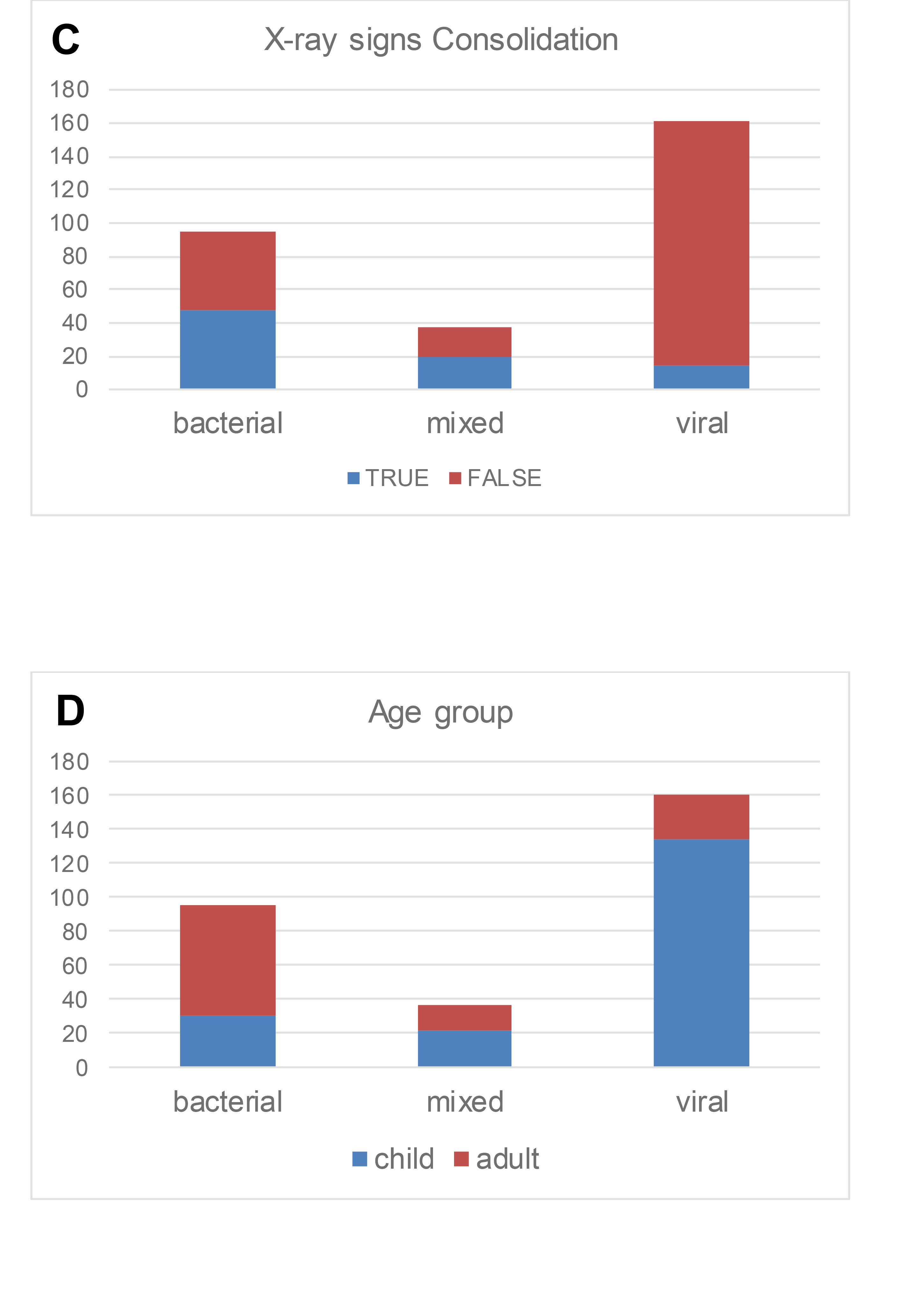

Supplement: S2 Fig — (A) C-reactive protein (CRP). (B) Absolute neutrophil count (ANC). (C) X-ray signs Consolidation. (D) Age group. In (A) and (B), the mean and standard error of mean are shown. In (C) and (D), the number of patients per category is shown. The patients with mixed infection are indicated separately although they were calculated as possessing ‘bacterial infection’ class labels in this study. (ZIP) [file pone.0267140.s002.zip › S2_Fig_C-D.tif]
